# l-Talitol

**DOI:** 10.1107/S2414314626008667

**Published:** 2026-08-25

**Authors:** Tomoko Takashita, Yoji Horii, Shota Miyoshi, Tomohiko Ishii

**Affiliations:** a Graduate School of Science for Creative Emergence Kagawa University, 2217-20 Hayashi-cho Takamatsu Kagawa 761-0396 Japan; Katholieke Universiteit Leuven, Belgium

**Keywords:** crystal structure, hydrogen bonding, rare sugar, sugar alcohol, talitol

## Abstract

The title compound, C_6_H_14_O_6_, the sugar alcohol corresponding to l-talose, was crystallized from aqueous solution. Colourless block-shaped single crystals suitable for single-crystal X-ray diffraction analysis were obtained. The title compound crystallizes in the monoclinic space group *P*2_1_, with one mol­ecule in the asymmetric unit. In the crystal, all six hy­droxy groups act as donors in O—H⋯O hydrogen bonds, forming a three-dimensional hydrogen-bonded network. The crystal structure of its enanti­omer, d-altritol (d-talitol), has been reported previously [Kopf *et al.* (1991 ▸). *Carbohydr. Res.***217**, 1–6].

## Abstract 2

The crystal structure of l-talitol, the sugar alcohol corresponding to l-talose, is presented. The mol­ecules are linked by O—H⋯O hydrogen bonds into a three-dimensional network.

## Graphical abstract

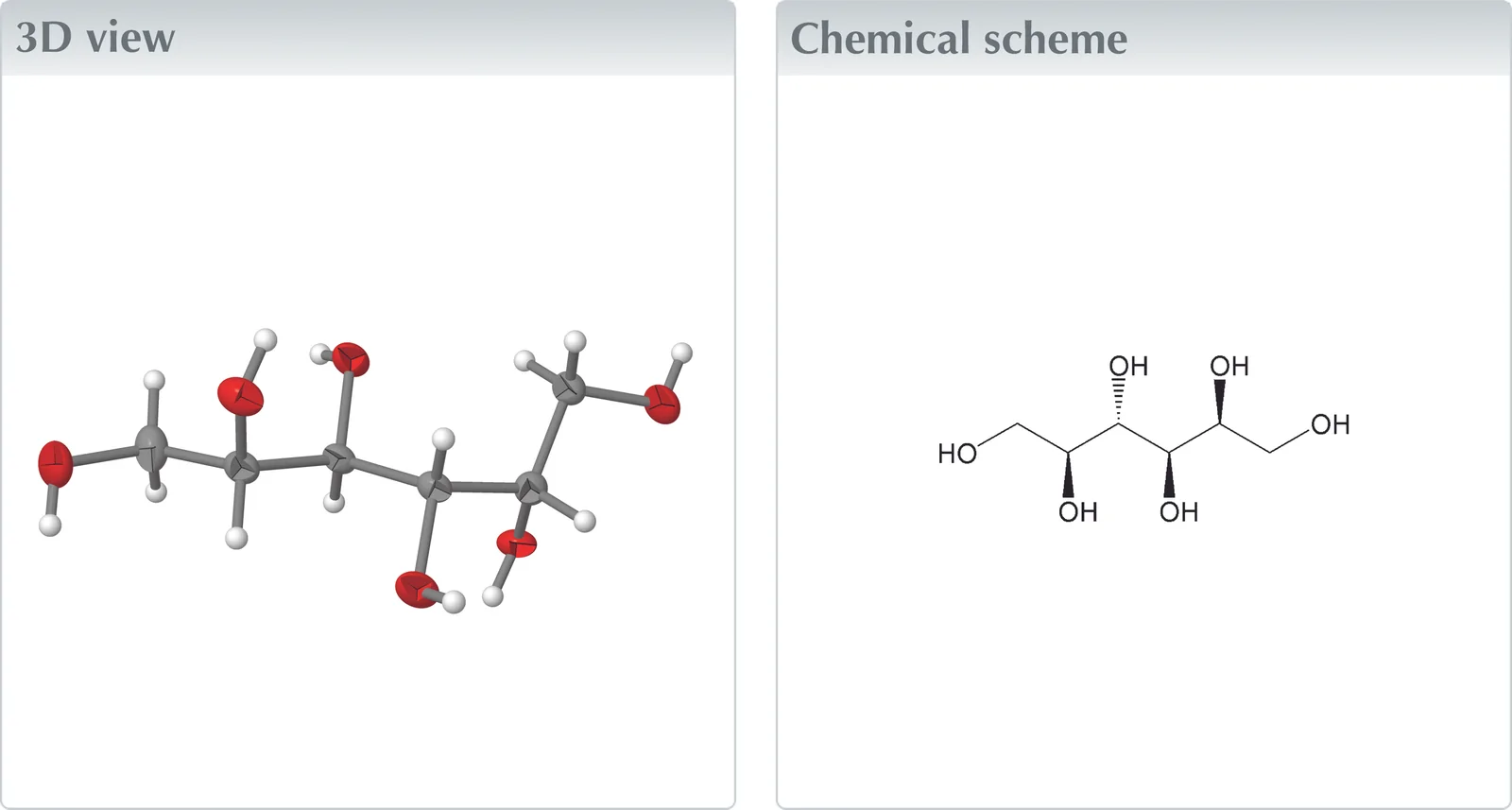

## Structure description

l-Talitol is the sugar alcohol corresponding to the rare sugar l-talose. Systematic bioproduction strategies have expanded the availability of rare hexoses and their corresponding sugar alcohols (Izumori, 2002 ▸). Sugar alcohols possess multiple hy­droxy groups and can therefore form extensive inter­molecular hydrogen-bonding networks. Elucidation of their mol­ecular conformations and crystal packing is important for understanding their solid-state properties.

The crystal structure of the enanti­omer, d-altritol (d-talitol), was reported previously by Kopf *et al.* (1991 ▸; CSD refcode JOJZOX). The present study provides a modern single-crystal structure determination of the corresponding *L* enanti­omer using Cu *K*α radiation. Compared with the earlier determination, for which a conventional *R* value of 0.054 was reported, the present refinement gives a lower value of *R*_1_ = 0.0292 and unit-cell parameters with smaller standard uncertainties. Furthermore, the anomalous-scattering data permitted assessment of the assigned absolute configuration, giving a Flack parameter of 0.07 (16) based on 570 quotients. The present determination therefore provides direct crystallographic characterization of l-talitol and a more precise modern dataset for this enanti­omorphic pair.

**Figure 1:**
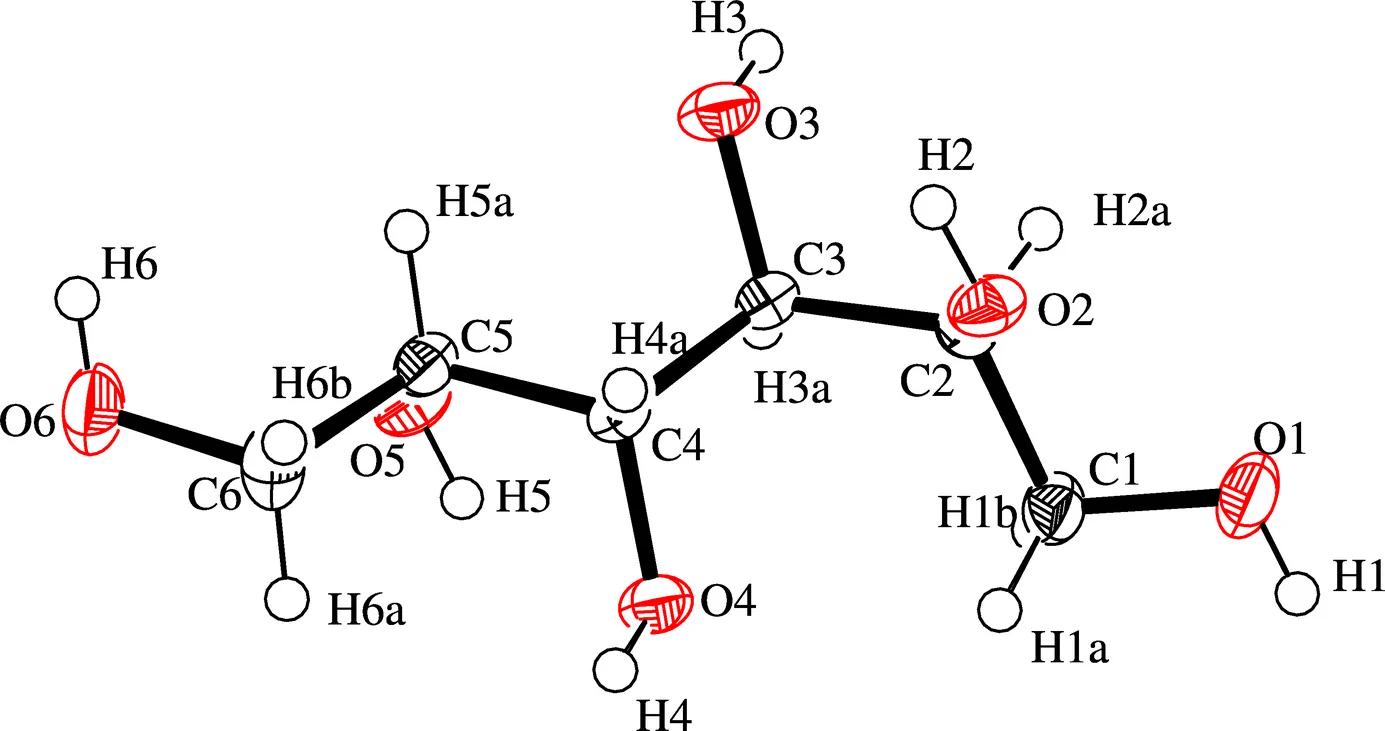
Mol­ecular structure of l-talitol, showing the atom-labelling scheme. Displacement ellipsoids are drawn at the 50% probability level, and hydrogen atoms are shown as spheres of arbitrary radii.

The title compound, C_6_H_14_O_6_ (Fig. 1 ▸), adopts an acyclic six-carbon chain structure. Single-crystal X-ray diffraction analysis revealed that it crystallizes in the monoclinic space group *P*2_1_. The asymmetric unit contains one mol­ecule of l-talitol.

**Figure 2:**
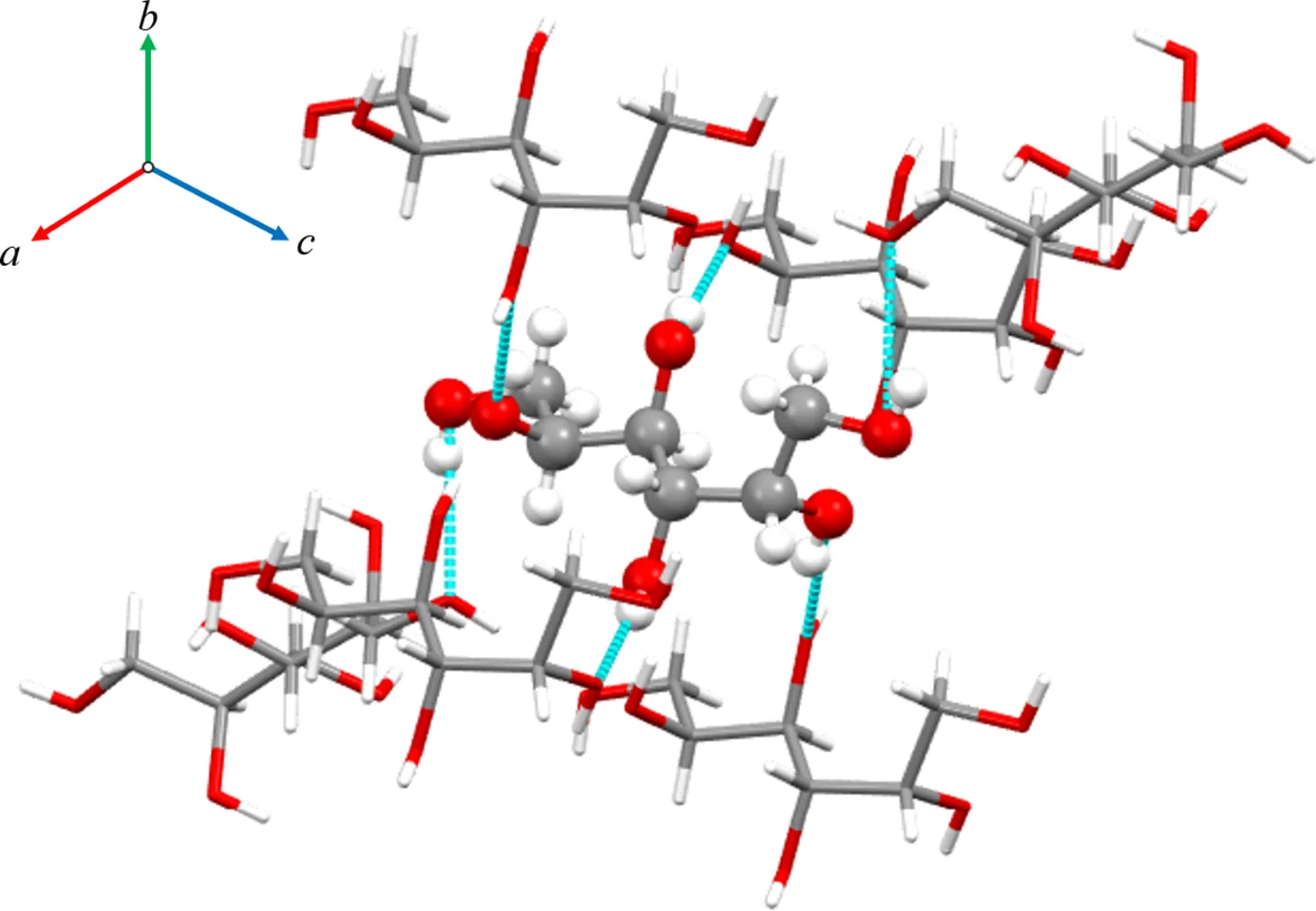
Crystal packing of l-talitol, showing part of the three-dimensional hydrogen-bonded network, with the unit-cell directions indicated. The cyan dashed lines indicate the O⋯O contacts associated with the O—H⋯O hydrogen bonds. The central mol­ecule is shown using a ball-and-stick representation, whereas the surrounding mol­ecules are shown using a capped-stick representation.

In the crystal, all six hy­droxy groups act as hydrogen-bond donors (Table 1 ▸), all of which are intermolecular interactions. These inter­actions link the mol­ecules in all three crystallographic directions, producing a three-dimensional hydrogen-bonded network, as illustrated in Fig. 2 ▸.

## Synthesis and crystallization

Commercially available l-talitol [Tokyo Chemical Industry Co., Ltd. (TCI)] was used as received without further purification. The sample was dissolved in water, and the solution was allowed to evaporate slowly at room temperature. Colorless block-shaped single crystals suitable for single-crystal X-ray diffraction analysis were obtained.

## Refinement

Crystal data, data collection and structure refinement details are summarized in Table 2 ▸. The absolute configuration was assigned on the basis of the known configuration of the commercially available l-talitol sample and was further supported by the refined Flack parameter. The Flack parameter was 0.07 (16), determined using 570 quotients of the type [(*I*^+^) − (*I*^−^)]/[(*I*^+^) + (*I*^−^)] (Parsons *et al.*, 2013 ▸).

## Supplementary Material

Crystal structure: contains datablock(s) I. DOI: 10.1107/S2414314626008667/vm4082sup1.cif

Structure factors: contains datablock(s) I. DOI: 10.1107/S2414314626008667/vm4082Isup2.hkl

Point-by-point response to the Co-editors comments for the revised data article vm4082 (L-talitol), including a summary of the revisions and CCDC deposition number 2577672. DOI: 10.1107/S2414314626008667/vm4082sup3.docx

CCDC reference: 2577672

Additional supporting information:  crystallographic information; 3D view; checkCIF report

## Figures and Tables

**Table 1:** Hydrogen-bond geometry (Å, °)

*D*—H⋯*A*	*D*—H	H⋯*A*	*D*⋯*A*	*D*—H⋯*A*
O1—H1⋯O1^i^	0.82	2.28	2.9570 (18)	140
O2—H2⋯O4^ii^	0.82	1.99	2.747 (2)	154
O3—H3⋯O2^iii^	0.82	1.88	2.700 (2)	174
O4—H4⋯O5^iv^	0.82	1.89	2.7027 (19)	174
O5—H5⋯O3^v^	0.82	2.05	2.773 (2)	147
O6—H6⋯O6^vi^	0.82	2.16	2.9502 (19)	162

Symmetry codes: (i) 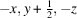; (ii) 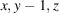; (iii) 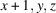; (iv) 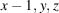; (v) 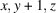; (vi) 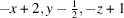.

**Table 2:** Experimental details

Crystal data	
Chemical formula	C_6_H_14_O_6_
*M*_r_	182.17
Crystal system, space group	Monoclinic, *P*2_1_
Temperature (K)	296
*a*, *b*, *c* (Å)	4.8901 (3), 5.1671 (3), 16.3657 (10)
β (°)	100.136 (4)
*V* (Å^3^)	407.07 (4)
*Z*	2
Radiation type	Cu *K*α
μ (mm^−1^)	1.17
Crystal size (mm)	0.10 × 0.10 × 0.10

Data collection	
Diffractometer	Rigaku R-AXIS RAPID
Absorption correction	Multi-scan (*ABSCOR*; Rigaku, 1995 ▸)
*T*_min_, *T*_max_	0.711, 1.000
No. of measured, independent and observed [*I* > 2σ(*I*)] reflections	4278, 1446, 1399
*R*_int_	0.059
(sin θ/λ)_max_ (Å^−1^)	0.602

Refinement	
*R*[*F*^2^ > 2σ(*F*^2^)], *wR*(*F*^2^), *S*	0.029, 0.075, 1.08
No. of reflections	1446
No. of parameters	116
No. of restraints	1
H-atom treatment	H-atom parameters constrained
Δρ_max_, Δρ_min_ (e Å^−3^)	0.20, −0.12
Absolute structure	Flack *x* determined using 570 quotients [(*I*^+^)−(*I*^−^)]/[(*I*^+^)+(*I*^−^)] (Parsons *et al.*, 2013 ▸)
Absolute structure parameter	0.07 (16)

Computer programs: *RAPID-AUTO* (Rigaku, 2009 ▸), *SHELXT2018/2* (Sheldrick, 2015*a* ▸), *SHELXL2018/3* (Sheldrick, 2015*b* ▸) and *OLEX2* 1.3 (Dolomanov *et al.*, 2009 ▸).

## full crystallographic data

### L-Talitol Crystal data

**Table:**

C_6_H_14_O_6_	*F*(000) = 196
*M**_r_* = 182.17	*D*_x_ = 1.486 Mg m^−^^3^
Monoclinic, *P*2_1_	Cu *K*α radiation, λ = 1.54187 Å
*a* = 4.8901 (3) Å	Cell parameters from 1932 reflections
*b* = 5.1671 (3) Å	θ = 5.5–68.2°
*c* = 16.3657 (10) Å	µ = 1.17 mm^−^^1^
β = 100.136 (4)°	*T* = 296 K
*V* = 407.07 (4) Å^3^	Block, clear light colourless
*Z* = 2	0.1 × 0.1 × 0.1 mm

### L-Talitol Data collection

**Table:**

Rigaku R-AXIS RAPID diffractometer	1399 reflections with *I* > 2σ(*I*)
ω scans	*R*_int_ = 0.059
Absorption correction: multi-scan (ABSCOR; Rigaku, 1995)	θ_max_ = 68.2°, θ_min_ = 5.5°
*T*_min_ = 0.711, *T*_max_ = 1.000	*h* = −5→5
4278 measured reflections	*k* = −6→6
1446 independent reflections	*l* = −19→19

### L-Talitol Refinement

**Table:**

Refinement on *F*^2^	H-atom parameters constrained
Least-squares matrix: full	*w* = 1/[σ^2^(*F*_o_^2^) + (0.0295*P*)^2^ + 0.0407*P*] where *P* = (*F*_o_^2^ + 2*F*_c_^2^)/3
*R*[*F*^2^ > 2σ(*F*^2^)] = 0.029	(Δ/σ)_max_ < 0.001
*wR*(*F*^2^) = 0.075	Δρ_max_ = 0.20 e Å^−^^3^
*S* = 1.08	Δρ_min_ = −0.12 e Å^−^^3^
1446 reflections	Extinction correction: SHELXL-2018/3 (Sheldrick, 2015b), Fc^*^=kFc[1+0.001xFc^2^λ^3^/sin(2θ)]^-1/4^
116 parameters	Extinction coefficient: 0.027 (4)
1 restraint	Absolute structure: Flack *x* determined using 570 quotients [(*I*^+^)-(*I*^-^)]/[(*I*^+^)+(*I*^-^)] (Parsons *et al.*, 2013)
Primary atom site location: dual	Absolute structure parameter: 0.07 (16)
Hydrogen site location: inferred from neighbouring sites	

### L-Talitol Special details

**Table:**

Geometry. All esds (except the esd in the dihedral angle between two l.s. planes) are estimated using the full covariance matrix. The cell esds are taken into account individually in the estimation of esds in distances, angles and torsion angles; correlations between esds in cell parameters are only used when they are defined by crystal symmetry. An approximate (isotropic) treatment of cell esds is used for estimating esds involving l.s. planes.
Refinement. All non-hydrogen atoms were refined anisotropically. Hydroxy H atoms were placed in geometrically calculated positions and treated as rotating groups, with O—H = 0.82 Å. C-bound H atoms were placed in calculated positions and refined using riding models, with C—H = 0.98 Å for CH groups and 0.97 Å for CH_2_ groups. For all H atoms, *U*_iso_(H) was constrained to 1.2*U*_eq_ of the parent C or O atom.

### L-Talitol Fractional atomic coordinates and isotropic or equivalent isotropic displacement parameters (Å^2^)

**Table:**

	*x*	*y*	*z*	*U*_iso_*/*U*_eq_	
O1	0.1487 (4)	0.2244 (3)	0.00353 (9)	0.0387 (5)	
H1	0.049895	0.334701	−0.022940	0.046*	
O2	0.2665 (3)	−0.0647 (3)	0.14835 (9)	0.0251 (4)	
H2	0.351707	−0.178860	0.176956	0.030*	
O3	0.8391 (3)	−0.0087 (3)	0.23126 (9)	0.0287 (4)	
H3	0.960388	−0.023251	0.202749	0.034*	
O4	0.3808 (3)	0.5215 (3)	0.25422 (8)	0.0260 (4)	
H4	0.256058	0.531070	0.281637	0.031*	
O5	0.9447 (3)	0.5638 (3)	0.33441 (9)	0.0277 (4)	
H5	0.849284	0.676689	0.308092	0.033*	
O6	0.8543 (4)	0.4580 (4)	0.49996 (9)	0.0400 (5)	
H6	0.939124	0.322089	0.511232	0.048*	
C1	0.2905 (5)	0.3341 (5)	0.07904 (12)	0.0268 (5)	
H1A	0.158245	0.404113	0.110957	0.032*	
H1B	0.411851	0.472857	0.067510	0.032*	
C2	0.4582 (4)	0.1203 (4)	0.12684 (12)	0.0223 (5)	
H2A	0.563950	0.035165	0.088863	0.027*	
C3	0.6663 (4)	0.2077 (4)	0.20298 (12)	0.0205 (4)	
H3A	0.781593	0.347156	0.186723	0.025*	
C4	0.5431 (4)	0.2952 (4)	0.27779 (11)	0.0202 (4)	
H4A	0.422927	0.158257	0.293084	0.024*	
C5	0.7699 (4)	0.3574 (4)	0.35239 (12)	0.0229 (5)	
H5A	0.885797	0.202833	0.364967	0.027*	
C6	0.6458 (5)	0.4218 (6)	0.42847 (12)	0.0326 (5)	
H6A	0.535703	0.578391	0.418094	0.039*	
H6B	0.523187	0.282646	0.438795	0.039*	

### L-Talitol Atomic displacement parameters (Å^2^)

**Table:**

	*U*^11^	*U*^22^	*U*^33^	*U*^12^	*U*^13^	*U*^23^
O1	0.0476 (11)	0.0336 (10)	0.0273 (8)	0.0020 (9)	−0.0144 (7)	0.0010 (7)
O2	0.0235 (8)	0.0178 (7)	0.0326 (8)	0.0016 (6)	0.0010 (6)	0.0039 (6)
O3	0.0253 (8)	0.0309 (9)	0.0311 (8)	0.0116 (7)	0.0082 (6)	0.0070 (7)
O4	0.0250 (8)	0.0290 (9)	0.0250 (7)	0.0096 (7)	0.0073 (6)	0.0054 (6)
O5	0.0195 (7)	0.0287 (9)	0.0336 (8)	0.0001 (7)	0.0007 (6)	0.0071 (7)
O6	0.0508 (11)	0.0401 (10)	0.0238 (7)	0.0001 (9)	−0.0080 (7)	−0.0046 (7)
C1	0.0335 (12)	0.0224 (10)	0.0222 (9)	−0.0014 (10)	−0.0011 (8)	0.0014 (9)
C2	0.0228 (10)	0.0227 (10)	0.0217 (9)	0.0010 (9)	0.0043 (8)	−0.0004 (8)
C3	0.0191 (10)	0.0203 (10)	0.0215 (9)	0.0022 (8)	0.0015 (8)	0.0033 (8)
C4	0.0198 (10)	0.0203 (10)	0.0199 (8)	0.0028 (8)	0.0022 (7)	0.0042 (8)
C5	0.0225 (10)	0.0219 (11)	0.0227 (9)	0.0007 (8)	−0.0004 (8)	0.0028 (8)
C6	0.0327 (13)	0.0417 (13)	0.0222 (10)	−0.0032 (11)	0.0018 (9)	−0.0033 (10)

### L-Talitol Geometric parameters (Å, º)

**Table:**

O1—H1	0.8200	C1—H1B	0.9700
O1—C1	1.424 (2)	C1—C2	1.510 (3)
O2—H2	0.8200	C2—H2A	0.9800
O2—C2	1.425 (3)	C2—C3	1.532 (3)
O3—H3	0.8200	C3—H3A	0.9800
O3—C3	1.429 (2)	C3—C4	1.525 (3)
O4—H4	0.8200	C4—H4A	0.9800
O4—C4	1.428 (3)	C4—C5	1.532 (2)
O5—H5	0.8200	C5—H5A	0.9800
O5—C5	1.429 (2)	C5—C6	1.515 (3)
O6—H6	0.8200	C6—H6A	0.9700
O6—C6	1.423 (2)	C6—H6B	0.9700
C1—H1A	0.9700		
			
C1—O1—H1	109.5	C2—C3—H3A	109.1
C2—O2—H2	109.5	C4—C3—C2	116.15 (16)
C3—O3—H3	109.5	C4—C3—H3A	109.1
C4—O4—H4	109.5	O4—C4—C3	107.77 (14)
C5—O5—H5	109.5	O4—C4—H4A	109.3
C6—O6—H6	109.5	O4—C4—C5	109.48 (17)
O1—C1—H1A	110.3	C3—C4—H4A	109.3
O1—C1—H1B	110.3	C3—C4—C5	111.66 (15)
O1—C1—C2	107.18 (18)	C5—C4—H4A	109.3
H1A—C1—H1B	108.5	O5—C5—C4	111.58 (16)
C2—C1—H1A	110.3	O5—C5—H5A	107.9
C2—C1—H1B	110.3	O5—C5—C6	110.23 (18)
O2—C2—C1	107.33 (17)	C4—C5—H5A	107.9
O2—C2—H2A	107.4	C6—C5—C4	111.25 (17)
O2—C2—C3	111.62 (16)	C6—C5—H5A	107.9
C1—C2—H2A	107.4	O6—C6—C5	111.83 (19)
C1—C2—C3	115.35 (17)	O6—C6—H6A	109.3
C3—C2—H2A	107.4	O6—C6—H6B	109.3
O3—C3—C2	107.65 (16)	C5—C6—H6A	109.3
O3—C3—H3A	109.1	C5—C6—H6B	109.3
O3—C3—C4	105.46 (14)	H6A—C6—H6B	107.9
			
O1—C1—C2—O2	−65.3 (2)	O5—C5—C6—O6	−60.8 (3)
O1—C1—C2—C3	169.64 (19)	C1—C2—C3—O3	−169.67 (16)
O2—C2—C3—O3	67.5 (2)	C1—C2—C3—C4	72.4 (2)
O2—C2—C3—C4	−50.4 (2)	C2—C3—C4—O4	−64.3 (2)
O3—C3—C4—O4	176.62 (16)	C2—C3—C4—C5	175.45 (19)
O3—C3—C4—C5	56.4 (2)	C3—C4—C5—O5	61.3 (2)
O4—C4—C5—O5	−57.9 (2)	C3—C4—C5—C6	−175.13 (18)
O4—C4—C5—C6	65.6 (2)	C4—C5—C6—O6	174.9 (2)

### L-Talitol Hydrogen-bond geometry (Å, º)

**Table:**

*D*—H···*A*	*D*—H	H···*A*	*D*···*A*	*D*—H···*A*
O1—H1···O1^i^	0.82	2.28	2.9570 (18)	140
O2—H2···O4^ii^	0.82	1.99	2.747 (2)	154
O3—H3···O2^iii^	0.82	1.88	2.700 (2)	174
O4—H4···O5^iv^	0.82	1.89	2.7027 (19)	174
O5—H5···O3^v^	0.82	2.05	2.773 (2)	147
O6—H6···O6^vi^	0.82	2.16	2.9502 (19)	162

Symmetry codes: (i) −*x*, *y*+1/2, −*z*; (ii) *x*, *y*−1, *z*; (iii) *x*+1, *y*, *z*; (iv) *x*−1, *y*, *z*; (v) *x*, *y*+1, *z*; (vi) −*x*+2, *y*−1/2, −*z*+1.
