# Supplementary material for: l-Talitol
Source: IUCrdata. 2026 Aug 25;11(Pt 8):x260866. doi: 10.1107/S2414314626008667 (PMC13548380; doi:10.1107/S2414314626008667)
Supplement: Supplementary file 1 [file x-11-x260866-sup3.docx]

Response to Co-editor Professor L. Van Meervelt

Title: L-talitol

Manuscript ID: vm4082

CCDC deposition number: 2577672

Dear Professor Van Meervelt,

We sincerely thank you for your careful assessment of our data article and for your helpful comments. In accordance with your suggestions, we have revised the manuscript to clarify the significance of reporting the *L* enantiomer, expanded the description of the hydrogen-atom refinement and revised Figure 2 to include the unit-cell directions. Our point-by-point responses are provided below.

The crystallographic data for the title compound have been deposited with the Cambridge Crystallographic Data Centre under CCDC deposition number 2577672.

**Comment 1:**

The authors need to provide a better justification why they are publishing an enantiomer of a known structure; presumably the precision is higher than the 1991 study (other wavelength, Flack...)?

**Response:**

The manuscript has been revised to provide a clearer justification for reporting the crystal structure of *L*-talitol. The previously reported structure of the enantiomer, *D*-altritol (*D*-talitol; CSD refcode JOJZOX), is now explicitly cited and compared with the present determination. The present study uses Cu Kα radiation, gives a lower conventional residual and unit-cell parameters with smaller standard uncertainties than the 1991 determination, and provides an assessment of the assigned absolute configuration through the Flack parameter. These points have been added to the manuscript to clarify the significance of the present crystallographic study.

**Comment 2:**

Line 36: add C-H and O-H distances and constraints used for U(H)

**Response:**

The refinement section has been revised to specify the constrained hydrogen-atom treatment. The O—H distances were fixed at 0.82 Å, the C—H distances at 0.98 Å for CH groups and 0.97 Å for CH₂ groups, and Uiso(H) was set to 1.2Ueq of the corresponding parent atom. Hydroxy H atoms were treated as rotating groups, while C-bound H atoms were refined using riding models.

**Comment 3:**

Figure 2: add unit cell directions.

**Response:**

Figure 2 has been revised to include the *a*, *b* and *c* unit-cell directions. The figure caption has also been revised to describe the unit-cell directions, hydrogen bonds and molecular representations.

We hope that these revisions have satisfactorily addressed all of the comments and that the revised data article is now suitable for publication in IUCrData.

Yours sincerely,

Tomoko Takashita

Graduate School of Science for Creative Emergence, Kagawa University, Japan
